# Editorial: Cervical cancer awareness – volume I: screening, vaccines, and prevention

**DOI:** 10.3389/frph.2025.1579018

**Published:** 2025-05-12

**Authors:** Anayawa Nyambe, Mwansa Ketty Lubeya

**Affiliations:** ^1^ Strategic Center for Health Systems Metrics & Evaluations (SCHEME), School of Public Health, University of Zambia, Lusaka, Zambia; ^2^ Department of Obstetrics and Gynaecology, School of Medicine, University of Zambia, Lusaka, Zambia

**Keywords:** cervical cancer, precursors, barriers, awareness, HPV vaccination, HPV testing, screening, prevention

**Editorial on the Research Topic**
Cervical cancer awareness – volume I: screening, vaccines, and prevention

## Introduction

Cervical cancer remains the fourth most common cancer among women worldwide. Low- and middle-income countries experience the highest burden due to limited access to HPV vaccination, screening, and treatment, leading to high morbidity and mortality.

This first volume of Cervical Cancer Awareness: Screening, Vaccination, and Prevention, supports the World Health Organization's (1) goal of eliminating cervical cancer defined as incidence of 4/100,000 women. It features articles on HPV self-sampling, DNA methylation assays, patient navigation and immunotherapy. Studies include scoping reviews of high-risk populations such as female sex workers, national and regional epidemiological studies, clinical protocols and molecular investigations to improve diagnosis and care.

These studies provide evidence-based insights needed for targeted and culturally responsive healthcare strategies to reduce the cervical cancer burden and enhance the quality of life for all populations.

## Cervical cancer screening, vaccines and prevention

This section presents brief overviews of the featured articles, each contributing unique insights into aspects of cervical cancer.

Addressing the needs of high-risk populations, Vimpere et al. offer a global scoping review of 13 studies (1989–2021) on cervical cancer screening programs for female sex workers (FSWs), focussing on feasibility strategies, sustainability, and challenges. Unlike previous research that largely centres on STIs or HIV, this review analyses interventions tailored for FSWs. It identifies effective strategies like the “Screen and Treat” approach, HPV-DNA self-sampling, and integration of FSW-targeted services into STI services. Major challenges included screening follow-up loss rates of 35%–60% and the lack of long-term sustainability. The review emphasizes the need for tailored cervical cancer programs for FSWs to reduce disease burden and meet global elimination goals.

Using a nationally representative dataset, Zhao et al. found an inverse relationship between income level and HPV infection in American women. Higher-income groups showed significantly lower infection rates. The novelty of this study lies in its examination of the relationship between the family income-to-poverty ratio (PIR), and HPV infection prevalence in American women aged 20 and older. Adding depth to previous studies by quantifying and stratifying PIR's effect across demographic and health subgroups, such as age, race, and sexual activity. The results emphasise addressing socioeconomic disparities to improve HPV prevention strategies around HPV screening and vaccine accessibility in low-income populations.

Mboineki et al., systematically evaluate the training and impact of patient navigators in promoting screening uptake among 202 Tanzanian women, where screening rates remain low despite free services. Unlike previous studies, it addresses the preparation of patient navigators and their influence on women's knowledge, awareness, beliefs, intentions, and screening uptake within a culturally relevant context. The study suggests that women may participate in screening due to education, social influence and follow-up by navigators. Additionally, it emphasises the importance of remuneration for patient navigators, proposing a sustainable model for improved CCS engagement.

The study by Tenkir et al., documents the prevalence of precancerous cervical lesions (14%) and suspected cancer (4.3%) in Southern Ethiopia, and identifies key risk factors including early sexual activity, multiple partners, HIV, and STIs. Unlike prior studies that largely focused on HIV-positive women or urban populations in Northern and Central Ethiopia, this study targets urban, rural, and pastoralist women in an underrepresented region. Its findings reveal regional disparities and underline the urgent need for targeted prevention strategies, including earlier HPV vaccination, improved STI management, and expanded screening coverage. This study strengthens the case for targeted prevention and screening in similar low-resource settings.

Zhong et al., genotyped HPV in 61,422 Chinese women with histologically confirmed cervical diagnoses, revealing differences from Western cohorts and gaps in current vaccine coverage. Results showed high-risk HPV (hrHPV) positivity rates of 55.1% overall, which were highest in cervical cancers (93.7%–94.1%). HPV16 was the most common genotype, followed by HPV52 and HPV58, with HPV18 more prevalent in glandular lesions. Indicating that current vaccines offer partial protection (69.1%–85.8%). This study provides crucial epidemiological data to inform screening strategies, public health policies, and the development of vaccines targeting additional hrHPV types.

Unlike previous studies limited to hrHPV-positive populations, Kong et al., evaluated DNA methylation in 307 patients, including hrHPV-negative cases, post-treatment residual disease, and endometrial carcinomas. Methylation assays were demonstrated to have lower sensitivity but higher specificity compared to traditional hrHPV testing, with favorable results for CIN2+, residual lesions, and cervical adenocarcinoma. Notably, methylation was found to be a potential prognostic biomarker for residual disease and endometrial carcinomas, areas previously underexplored. These findings support DNA methylation testing as a complementary and independent screening method, especially in settings with limited cytology or hrHPV testing access.

Li et al., provide a review of anti-PD-1 immunotherapy combined with other treatments, offering insights that can improve the effectiveness of advanced cervical cancer therapies. This review is unique because it analyses ongoing and published trials and potential molecular biological mechanisms that investigate anti-PD-1 both alone and in combination with chemotherapy, radiotherapy, targeted therapy, and dual immunotherapy (e.g., anti-CTLA-4), across various stages of cervical cancer. This study not only reinforces the role of anti-PD-1 as a treatment option for recurrent or metastatic and locally advanced cervical cancer but also provides direction for future clinical strategies.

Finally, Cong et al., establish evidence-based, cervical transformation zone (TZ) guidelines for optimal excision lengths during LEEP procedures, which improve treatment outcomes and surgical precision. Unlike previous studies that provided broad or inconsistent recommendations, this study of 618 women determined that a length of 10–15 mm was sufficient for TZ1 and TZ2, while 17–25 mm was optimal for TZ3. Risk factors for positive margins included age and high-grade squamous intraepithelial lesions. This targeted approach improves clinical decision-making and also adds to literature by covering a topic that has lacked standardized guidelines despite being critical for effective treatment.

## Closing remarks

The studies in this collection reinforce that cervical cancer is preventable and manageable. Eliminating it requires comprehensive and context-specific strategies for diverse populations. Interventions must be innovative, inclusive, and responsive to social, cultural, and economic contexts through better diagnostic tools, targeted community outreach, or policy-informed vaccine development.
